# A literature review of dopamine in binge eating

**DOI:** 10.1186/s40337-022-00531-y

**Published:** 2022-01-28

**Authors:** Yang Yu, Renee Miller, Susan W. Groth

**Affiliations:** 1 School of Nursing, University of Rochester, 601 Elmwood Avenue, Rochester, NY 14642 USA; 2 Brain and Cognitive Sciences, University of Rochester, 303F Meliora Hall, Rochester, NY 14627 USA

**Keywords:** Bing eating disorder, Bulimia nervosa, Neurophysiology, Hyperdopaminergic state, Hypodopaminergic state

## Abstract

**Objective:**

Binge eating, a core diagnostic symptom in binge eating disorder and bulimia nervosa, increases the risk of multiple physiological and psychiatric disorders. The neurotransmitter dopamine is involved in food craving, decision making, executive functioning, and impulsivity personality trait; all of which contribute to the development and maintenance of binge eating. The objective of this paper is to review the associations of dopamine levels/activities, dopamine regulator (e.g., dopamine transporter, degrading enzymes) levels/activities, and dopamine receptor availability/affinity with binge eating.

**Methods:**

A literature search was conducted in PubMed and PsycINFO to obtain human and animal studies published since 2010.

**Results:**

A total of 31 studies (25 human, six animal) were included. Among the human studies, there were 12 case–control studies, eight randomized controlled trials, and five cross-sectional studies. Studies used neuroimaging (e.g., positron emission tomography), genetic, and pharmacological (e.g., dopamine transporter inhibitor) techniques to describe or compare dopamine levels/activities, dopamine transporter levels/activities, dopamine degrading enzyme (e.g., catechol-O-methyltransferase) levels/activities, and dopamine receptor (e.g., D1, D2) availability/affinity among participants with and without binge eating. Most human and animal studies supported an altered dopaminergic state in binge eating (26/31, 83.9%); however, results were divergent regarding whether the altered state was hyperdopaminergic (9/26, 34.6%) or hypodopaminergic (17/26, 65.4%). The mixed findings may be partially explained by the variability in sample characteristics, study design, diagnosis criteria, and neuroimaging/genetic/pharmacological techniques used. However, it is possible that instead of being mutually exclusive, the hyperdopaminergic and hypodopaminergic state may co-exist, but in different stages of binge eating or in different individual genotypes.

**Conclusions:**

For future studies to clarify the inconsistent findings, a homogenous sample that controls for confounders that may influence dopamine levels (e.g., psychiatric diseases) is preferable. Longitudinal studies are needed to evaluate whether the hyper- and hypo-dopaminergic states co-exist in different stages of binge eating or co-exist in individual phenotypes.

**Plain Language Summary:**

Binge eating is characterized by eating a large amount of food in a short time and a feeling of difficulty to stop while eating. Binge eating is the defining symptom of binge eating disorder and bulimia nervosa, both of which are associated with serious health consequences. Studies have identified several psychological risk factors of binge eating, including a strong desire for food, impaired cognitive skills, and distinct personality traits (e.g., quick action without careful thinking). However, the physiological markers of binge eating remain unclear. Dopamine is a neurotransmitter that is heavily involved in feeding behavior, human motivation, cognitive ability, and personality. Therefore, dopamine is believed to play a critical role in binge eating. This review synthesized study findings related to the levels and activities of dopamine, dopamine regulators, and dopamine receptors in the context of binge eating. The primary finding is that most studies that used neuroimaging, genetic, or drug techniques found an altered dopaminergic state related to binge eating. However, the literature is inconsistent concerning the direction of the alteration. Considering the mixed findings and the limitations in study design, future studies, especially those that include repeated measurements, are needed to clarify the role of dopamine in binge eating.

## Introduction

Binge eating, a core diagnostic symptom in binge eating disorder (BED) and bulimia nervosa (BN), is characterized by eating a large amount of food in a short time and a sense of loss of control while eating [1]. Binge eating (with or without diagnosed BED or BN) affects 10–40% of children and adults [2, 3], and it is a strong predictor of obesity and increases the risk of multiple physiological (e.g., metabolic syndrome, malnutrition) and psychiatric (e.g., depression) disorders [4, 5]. Despite the high prevalence and negative consequences, many patients with binge eating remain undiagnosed and untreated [6]. Even among those who are diagnosed and receive the most validated cognitive-behavioral therapy, 60% of them fail to fully abstain from binge eating [7]. To facilitate early diagnosis and to inform the development of novel treatment strategies, there is a critical need to identify the biomarkers that are involved in the development and maintenance of binge eating.

Enhanced food craving, impaired decision making, diminished executive function, and impulsivity personality traits are among the main risk factors that drive or perpetuate binge eating. Food craving is an intense desire or motivation to consume food, and it has been consistently associated with more frequent or more severe binge eating behaviors in cross-sectional and prospective studies [8, 9]. Decision making is regulated by two distinct systems: goal-directed (flexible, behaviors are adjusted based on anticipated outcomes) and habitual (automatic, behaviors are based on previous learning). There is converging evidence that binge eating is associated with an imbalance between these two systems with a greater reliance on habitual control [10–12]. Executive function refers to a set of high-order cognitive abilities that allow a person to perform complex daily activities. A large body of literature has supported that deficits in the three main components of executive function, including working memory, inhibitory control, and cognitive flexibility, contribute to binge eating symptoms [13, 14]. Finally, impulsivity as a personality dimension is generally described as a tendency to engage in premature behaviors without sufficient consideration of possible consequences, and it has been strongly related to the loss of control experienced during binge eating episodes [15, 16].

Food craving, decision making, executive function, and impulsivity are regulated by distinguishable, although somewhat overlapping, brain regions and neurocircuitry. For example, a large brain network including the ventral tegmental area, ventral striatum (nucleus accumbens), lateral hypothalamus, orbitofrontal cortex, and amygdala is involved in the excursion of food craving. In contrast, the dorsal striatum, which can be further divided into dorsomedial striatum (caudate) and dorsolateral striatum (putamen), is essential for orchestrating goal-directed and habitual decision making [17, 18]. Furthermore, the prefrontal cortex, especially the lateral prefrontal cortex, is the major neural substrate of executive function. In terms of impulsivity, although its brain structural correlates have not been clarified, it is believed that various regions, including the striatum, prefrontal cortex, hippocampus, anterior cingulate cortex, temporal pole, and insula, are involved [15].

The neurotransmitter dopamine has attracted growing attention in the field of binge eating due to its widely distributed receptors in the brain regions and neurocircuitry implicated in food craving, decision making, executive function, and impulsivity, as well as its functional associations with these risk factors.

**Fig. 1:**
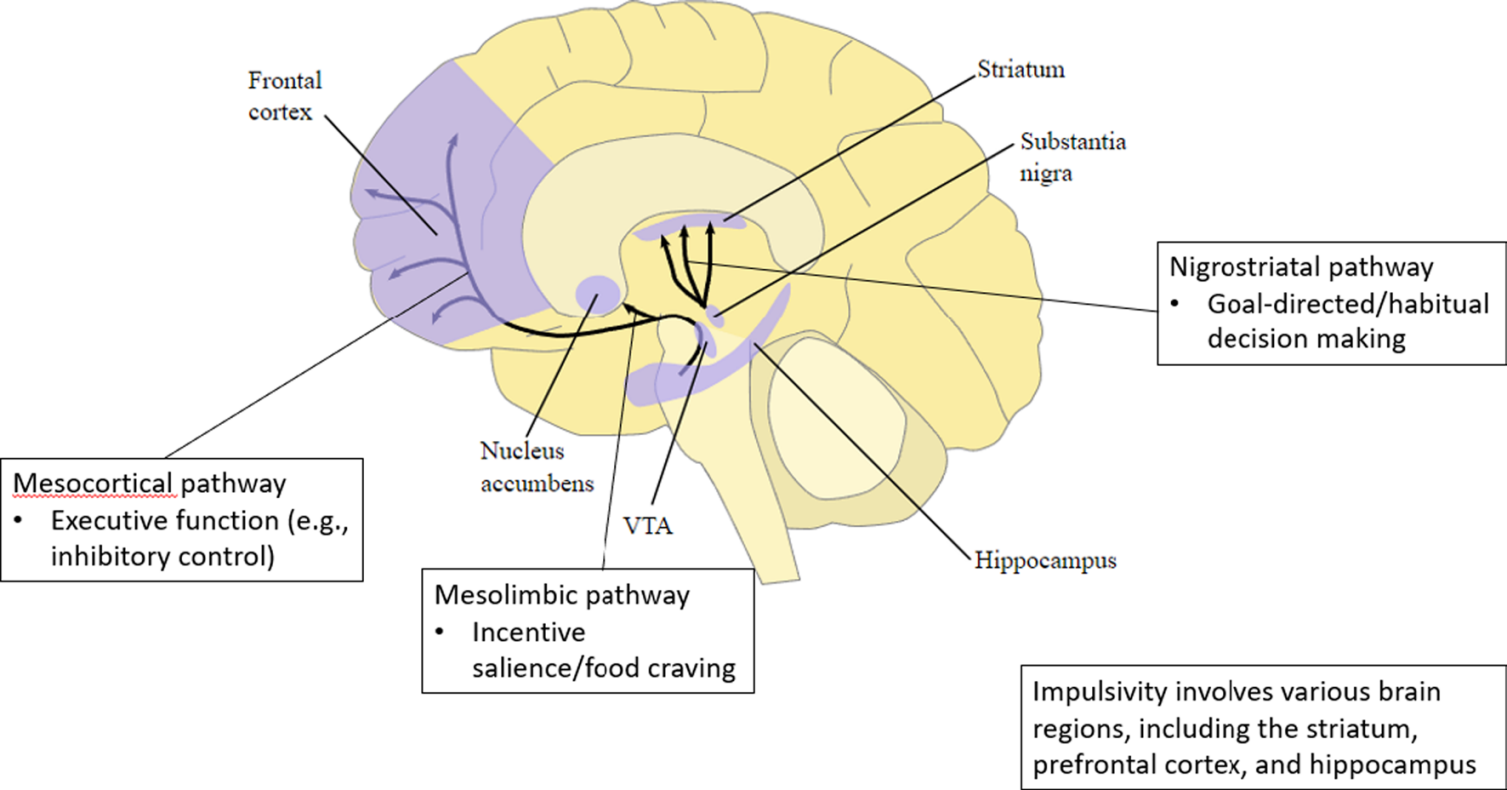
Dopaminergic pathways and corresponding risk factors of binge eating. * The original figure was developed by the National Institute of Health, and is in the public domain

Dopamine is synthesized and released by dopamine neurons located in three main areas in the midbrain: the ventral tegmental area, the substantia nigra, and the retrorubral field [19]. Dopamine neurons in the ventral tegmental area send projections to the ventral striatum (the main brain region relevant to food craving), forming the mesolimbic circuits [19]. The mesolimbic dopaminergic system has traditionally been associated with motivation. In the context of eating behaviors, the hyperactive mesolimbic dopaminergic system leads to an increased incentive salience or craving for food-related rewards, thus contributing to the initiation of food consumption [20–23]. In addition to sending projections to the ventral striatum, midbrain dopamine neurons in the ventral tegmental area further project to the prefrontal cortex (the main brain region responsible for executive function), via the mesocortical pathway. Dopamine in the prefrontal cortex serves as a neuromodulator that is essential for regulating inhibitory control, working memory, and set-shifting. For example, neuroimaging and pharmacological studies have provided evidence that dopamine agonists increase frontal cerebral blood flow, which is associated with better inhibitory control [24, 25]. Additionally, an inverted-U relationship of dopamine with working memory and set-shifting has been repeatedly reported such that too low or too high extracellular dopamine concentration in the prefrontal cortex can impair working memory and set-shifting [26–28]. In contrast, dopamine neurons in the substantia nigra send projections to the dorsal striatum (the key hub for the regulation of goal-directed and habitual decision making), forming the nigrostriatal circuits. Substantial evidence from animal studies has demonstrated that dopamine sensitization in the dorsal striatum accelerates the development of habit formation from previously goal-directed behaviors [29–31]. Finally, studies have found that a high magnitude of dopamine release or higher dopamine receptor capability in the striatum predicts higher levels of impulsivity in humans and animals [32, 33]. The major dopaminergic pathways and corresponding risk factors of binge eating are outlined in Fig. 1.

After being synthesized in the dopamine neurons and released into the synapse cleft, dopamine functions through binding to its receptors, generally distinct in two main subclasses: D1-like (D1 and D5 receptors) and D2-like (D2, D3, and D4 receptors) [34]. D1 and D2 receptors are abundant in the striatum and prefrontal cortex [34, 35], and they are the most studied in terms of regulating food craving, decision making, and executive functioning [36, 37].

The termination of dopamine function largely relies on dopamine clearance by the dopamine transporter, which drives the reuptake of extracellular dopamine into presynaptic neurons and consequently decreases the synaptic dopamine levels. Additionally, dopamine is degraded by enzymes such as monoamine oxidase and catechol-O-methyltransferase (COMT). COMT is most abundant in the prefrontal cortex and accounts for over 60% of the metabolic degradation of released dopamine in the prefrontal cortex [38]. While a detailed description of dopamine neurons, receptors, transporters, and degrading enzymes is beyond the scope of this paper, they have been extensively reviewed elsewhere [35, 39].

The role of dopamine in binge eating has been previously reviewed; however, these reviews were predominantly published between 2010 and 2015 [40–45]. Importantly, these reviews were either exclusively focused on animal models [40, 43] or included very few (< 5) human studies [41, 42, 44, 45]. Animal and human binge eating have similar manifestations, such as overeating in the absence of hunger and preference for high-energy foods, which suggest that they share overlapping biological mechanisms. However, animal models cannot fully replicate the complexity of human binge eating. For example, human binge eating is often triggered by psychological risk factors, while animal binge eating is manipulated by the experimenter. Therefore, despite that animal models represent a valuable tool, human studies are critical to better understand how dopamine contributes to binge eating. Furthermore, although previous reviews generally support dopamine alterations in BED or BN, the direction of the alterations appears to be mixed. Importantly, none of the reviews has attempted to reconcile the mixed results.

During the past decade, there have been increasing efforts to delineate the role of dopamine in binge eating both in humans and animals. Therefore, the goal of this review is to provide an updated assessment of the literature on binge eating and dopamine, including dopamine levels (synthesis, release), dopamine activities, dopamine regulator (dopamine transporter, degrading enzymes) levels/activities, and dopamine receptor availability/affinity, in both humans and animals.

## Methods

Animal studies were included if (1) they examined binge eating in relation to dopamine levels/activities, dopamine regulator levels/activities, or dopamine receptor availability/affinity, and (2) the binge eating was induced by one of the three standard paradigms: food restriction (periods of food restriction followed by periods of free access to palatable foods), food restriction and stress (periods of food restriction followed by stress exposure), and intermittent access (ad libitum access to standard chow and water, combined with intermittent access to palatable foods).

Human studies were included in this review if they (1) compared dopamine levels/activities, dopamine regulator levels/activities, or dopamine receptor availability/affinity between adults with binge eating, BED, or BN and healthy controls; or (2) described the associations of dopamine levels/activities, dopamine regulator levels/activities, or dopamine receptor availability/affinity with binge eating symptoms among healthy or community-based adults, or adults with binge eating, BED, or BN.

Qualitative studies, abstracts, editorials, case studies, book chapters, dissertation work, and review papers were excluded.

**Fig. 2:**
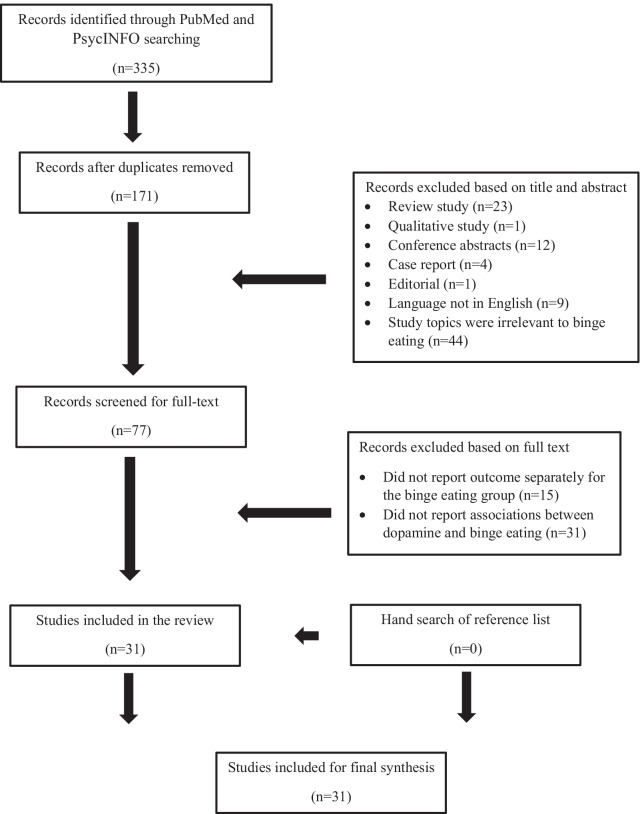
Study selection flow

The literature search was conducted in July 2020 with two databases (PubMed and PsycINFO) to obtain relevant studies published since 2010. The search included the combination of the following keywords: “dopamine”, “binge eating”, and “bulimia”. The database search was complemented by a hand search of the reference lists obtained from the identified articles. The study selection flow is presented in Fig. 2.

## Results

### Study characteristics

This review included 25 human studies and six animal studies. Among the 25 human studies, participants had BED (n = 6), BN (n = 7), remitted BN (n = 3), both BED and BN (n = 3), and binge eating symptoms (n = 3), and the remaining three studies were comprised of healthy adults or community-based adults. Most of the human studies used case–control design (n = 12), and the others were randomized controlled trials (RCTs, n = 7), cross-sectional studies (n = 5), and randomized crossover study (n = 1). For the six animal studies, four used male adult rats, and two used female adult rats. The intermittent access paradigm was consistently used to induce binge eating.

### Techniques used to measure dopamine levels/activities, dopamine regulator levels/activities, and dopamine receptor availability/affinity

#### Neuroimaging technique

##### PET

PET relies on the administration and subsequent detection of positron-emitting radiotracers. The radiotracer [^11^C]raclopride has a high selectivity and affinity to the D2 receptor; thus, it competes with endogenous dopamine binding to the D2 receptor—when endogenous dopamine increases, the radioligand signal decreases [46]. Furthermore, the change of radioligand signal before and after dopamine psychostimulant (e.g., methylphenidate) administration can be used to measure the extracellular dopamine release. Another radiotracer, [^18^F]fluorodopa, is an analog of a dopamine precursor, which is uptaken by the presynapse and consequently promotes dopamine synthesis. Therefore, PET can accomplish three primary goals: measuring dopamine receptor availability/affinity, measuring the extracellular dopamine release, and measuring the dopamine synthesis capacity. Three studies used PET in this review: two used [^11^C]raclopride in conjunction with methylphenidate to measure dopamine D2 receptor availability/affinity and dopamine release (after methylphenidate) [47, 48], and one used [^18^F]fluorodopa to measure dopamine synthesis capacity [49].

##### fMRI

fMRI measures the hemodynamic and metabolic consequences of brain neuronal activity known as blood-oxygen-level-dependent (BOLD) signal. Although the BOLD signal reflects a mix of neurotransmitter dynamics (e.g., dopamine, acetylcholine, serotonin), many studies have reported a significant relationship between dopamine levels/activities and BOLD signal variability in dopamine-relevant brain regions (e.g., striatum) [50, 51], thus supporting its use as an indirect measure of dopamine function. Among the three included fMRI studies, one measured the brain activity in response to taste stimuli, based on a dopamine-related reward learning paradigm, in several brain regions of interest (e.g., ventral putamen, lateral orbitofrontal cortex) [52]. The other two studies measured brain activity to money cues after dopamine depletion [53], or brain activity to food image after applying dopamine D3 antagonist [54]. The application of dopamine-related tasks or drugs in these studies further strengthened the confidence to use the BOLD signal as a robust proxy for dopamine function.

#### Genetic techniques

The two primary genetic approaches have been to measure gene expression levels and to look for naturally occurring genetic polymorphisms in dopaminergic genes. Three studies [55–57] analyzed mRNA expression for dopamine receptors and dopamine transporter, while the majority of studies examined the genetic polymorphisms coding for dopamine receptor D2 genes [58–61], receptor D3 genes [60], receptor D4 genes [61–63], dopamine transporter genes [59–61], and dopamine degrading enzyme COMT genes [59, 61, 64–66].

##### D2 receptor gene

Several polymorphisms of the D2 receptor gene have been studied: (1) Taq1A C/T: the T (A1) allele is associated with lower levels of D2 receptor availability and binding affinity relative to the C (A2) allele [67, 68]; (2) C957T: the T/T allele is associated with higher D2 receptor availability and binding affinity compared to C/T and C/C [69]; and (3) − 141 Ins/Del: the DelC minor allele is associated with reduced D2 expression [70].

##### D3 and D4 receptor genes

There are also polymorphisms in the lesser studied D3 and D4 receptors. The Ser9Gly variant is a functional polymorphic site in the D3 receptor gene, which increases the D3 receptor binding affinity for dopamine [71]. The D4 receptor contains a 48-base pair region that occurs with a variable number of tandem repeats in different individuals. This polymorphism is the most extensively investigated. Compared to the 2-repeat or 4-repeat allele, the 7-repeat (7R) allele decreases D4 receptor availability and binding affinity [72].

##### Dopamine transporter gene

The dopamine transporter gene also has a region with a variable number of tandem repeats, and it is the most studied dopamine transporter polymorphism. Relative to the 10-repeat allele, the 9-repeat allele is associated with lower dopamine transporter expression, resulting in increased synaptic dopamine levels for 9-repeat allele carriers [73].

##### COMT gene

The gene encoding the dopamine degrading enzyme, COMT, contains a well-studied polymorphism (Val/Met) that influences the protein’s ability to degrade extracellular dopamine. The Val allele has a 40% higher enzymatic activity than the Met allele; therefore, carriers of the Val/Val genotype degrade dopamine in the prefrontal cortex more efficiently, resulting in lower synaptic dopamine levels, compared to those with Met/Met or Val/Met genotype [74].

#### Pharmacological technique

In pharmacological studies, several drugs including the immediate dopamine precursor L-DOPA [75], dopamine synthesis inhibitor (alpha-methyl-para-tyrosine [53, 76, 77]), dopamine transporter inhibitors (i.e., methylphenidate [47, 48, 78]; lisdexamfetamine [79, 80]; dasotraline [81]), dopamine receptor agonists (i.e., D1 agonist SKF 81297 [56]; D2 agonist quinpirole [56]), and dopamine receptor antagonists (i.e., D1 receptor antagonist SCH 23390 [56, 82]; D2 receptor antagonist raclopride [82, 83]; D3 receptor antagonist GSK598809 [54]) have been used to manipulate the dopamine levels and activities.

### Studies that reported a hyperdopaminergic state in binge eating

The hyperdopaminergic state (n = 9) is characterized by (1) two human case–control studies that reported increased dopamine levels and higher dopamine receptor availability/affinity in patients with binge eating than those without binge eating; (2) five cross-sectional studies that showed positive associations of dopamine levels, activities, and receptor availability/affinity with binge eating symptoms among healthy adults, community-based adults, or adults with binge eating; and (3) two animal studies that demonstrated dopamine receptor-blocked or dopamine-depleted rats reduced or failed to develop binge eating symptoms (Table 1).

**Table 1:** Studies that reported a hyperdopaminergic state in binge eating

Study characteristics	Study purpose	Participant characteristics	Measurement	Results
Wang et al. 48 Case–control	Compare the brain dopamine responses to food stimuli between binge eaters and non-binge eaters	10 BED patients (8 female, 2 male; age: 38.5 ± 13.3; BMI: 43.4 ± 13.5) 8 obese controls (five female, 3 male; age: 41.8 ± 8.9; BMI: 36.5 ± 9.4) Exclusion: history of medical treatment for weight control, alcohol or drug abuse, neurological or psychiatric disorder, cardiovascular disease, diabetes, head trauma	PET and [11C]raclopride PET scanning was conducted in different conditions: placebo and methylphenidate under food intervention (view, smell, taste) or neutral intervention (pictures, toys, and clothing items)	Binge eaters had significantly more dopamine release than non-binge eater in caudate after methylphenidate under food intervention. However, the differences in putamen or in ventral striatum were not significant The increases of dopamine release across all subjects in caudate were correlated with binge eating severity
Nasser et al. 78 Cross-sectional	Assess dopamine response to oral food stimulation using electroretinographic Correlate dopamine response under placebo, methylphenidate and food stimuli with Binge Eating Scale and the Three Factor Eating Questionnaire	9 healthy, eating disorder-free adults (5 female, 4 male; age: 39 ± 10; BMI: 32 ± 5) Exclusion: diabetes, thyroid or renal disease, neurological disorders, Tourette’s syndrome, schizophrenia, bipolar disease, current major or situational depression, current or history of anorexia nervosa or bulimia nervosa, current use of any prescription medication, pregnancy, use of tobacco products or recreational drugs	Dopamine responses were assessed under four conditions: placebo (water), 10 mg methylpheidate, 20 mg methylphenidate, food stimulus	A significant increase in b-wave amplitude in response to the 20 mg methylphenidate dose and food stimuli Significant correlations between b-wave amplitude under the food stimulus condition and the Binge Eating Scale score
Davis et al. 58 Case–control	Compared five polymorphisms known to influence the function of the striatal dopamine D2 receptor between BED and controls The five polymorphisms: rs1800497[Taq1A], rs1799732 [− 141C ins/del], rs6277 [C957T], rs2283265, and rs12364283	79 BED obese adults (67 female; 12 male; age: 34.8 ± 6.5; BMI: 38.6 ± 7.2) 151 obese adults (104 female; 47 male; age: 35.6 ± 7.2; BMI: 38.7 ± 7.1) Exclusion: a current diagnosis of any psychotic disorder, substance abuse, alcoholism, or a serious medical/physical illness such as cancer	Non-enzymatic, high salt procedure was used to extract DNA from the whole blood	Compared to weight-matched controls, BED was significantly related to the rs1800497 and rs6277 genotypes that reflect enhanced striatal dopamine neurotransmission BED is distinguished by a greater density of D2 receptors and higher D2 binding potential compared to the obese controls The multi-locus D2 genetic profile observed in BED participants suggests enhanced dopamine signaling, and thereby increased striatal reactivity, compared to obese adults without binge eating
Davis et al. 59 Cross-sectional	Examine the associations between a multilocus genetic profile score and binge eating Multilocus genetic profile: Taq1A C/T, C957T, − 141 Ins/Del, DAT1, Val158Met	120 adults (82 female, 38 male), 24% of participants met the diagnostic criteria for BED Exclusion: a current diagnosis of any psychotic disorder, substance abuse, or a serious medical/physical illness such as cancer or heart disease	Non-enzymatic, high salt procedure was used to extract DNA from the whole blood Binge eating questionnaire	Higher multilocus genetic profile score (enhanced ventral striatum striatal dopamine-signaling) was associated with more bingeing
Donofry et al. 65 Cross-sectional	Test whether the COMT met allele increased risk for, and severity of, eating disorder symptomatology in community volunteers	1003 community-based Caucasian adults (female: 51.2%; age: 44.6 ± 6.8; BMI: 27.0 ± 5.4) Exclusion: a clinical history of neurologic illness, cardiovascular disease, cancer treatment within the previous year, schizophrenia, or other psychoses	DNA was isolated from white blood cells using the PureGene kit COMT val/met SNP was genotyped using florescence polarization Eating disorders inventory	Individuals carrying the met allele of the *COMT* val158met were 87% more likely than individuals with the val/val genotype to report symptoms on the Bulimia subscale It is possible that met allele carriers have greater difficulty exerting top-down control of behavior driven by midbrain dopamine activation
Gervasini et al. 62 Case–control	Determine dopamine Receptor D4 gene on general psychopathological symptoms in eating disorder patients	74 female with BN (age: 20.9 ± 8.1; BMI: 24.6 ± 6.9) 199 female with AN Exclusion: dementia, mental retardation, schizophrenia, Turner's syndrome, other neurological disorders and underlying endocrine pathologies	Genomic DNA purification was performed with a Qiagen blood midi kit Symptom Checklist 90 Revised	General psychopathological features such as somatization, obsessive–compulsive, anxiety, phobic anxiety, and paranoid ideation were significantly higher in BN women who carried haplotype DRD4*2 (non7R-L-C–C) DRD4 haplotypes may contribute to individual variance in personality features that predispose to disordered eating
Gonzalez et al. 60 Cross-sectional	Analyze the association between three common polymorphisms in the dopaminergic pathways with eating disorder symptoms in patients with BN or BED Three polymorphisms: DAT1 VNTR 10R/9R, DRD2 A2/A1 and DRD3 Ser9Gly	80 female with BN (BMI: 25.6 ± 8.9) 34 female with BED (BMI: 35.4 ± 11.5) Exclusion: neurological disorders (such as mental retardation, dementia or Turner syndrome) and underlying endocrine pathologies	Genomic DNA was isolated from whole blood samples using a standard phenol–chloroform extraction method Eating Disorders Inventory Test-2 (EDI-2) Revised Symptom Checklist 90 questionnaire	BED patients with the Ser9Gly variant showed higher EDI-2 scores than Ser9Ser carriers No associations were found for the BN group
Suarez-Ortiz et al. 83 Animal study	Determine the effect of D2 receptor antagonist on binge eating	Female Sprague Dawley rats Binge eating rats were induced by intermittent access to a sucrose solution Rats were divided into three groups: no access (control, n = 14), 2-h daily access (intermittent, n = 16), and 24-h daily access (ad libitum, n = 13) during 28 days Rats in the intermittent access group were randomly assigned to receive injections of vehicle or raclopride (n = 8)	Raclopride tartrate salt (Sigma Chemical Co., Toluca, Mexico) was dissolved in 0.9% saline solution, and was injected into nucleus accumbens	Blockade of dopamine D2 receptors in the nucleus accumbens prevented the effects of the intermittent access to the sucrose solution on meal frequency and duration Blockade of dopamine D2 receptors specifically decreased the sucrose solution intake
Mineo et al. 75 Animal study	Test whether dopamine manipulation using 6-hydroxydopamine (6-OHDA) and L-dopa will influence binge-like eating behavior	Male Wistar rats (n = 45) Sham-operated + saline (Sham; n = 15), parkinsonian 6-OHDA + saline (6-OHDA; n = 15), and 6-OHDA + L-dopa (L-dopa; n = 15) rats were randomly allotted into three groups: control group (standard chow), low restriction group, and high restriction group	L-dopa, 6–12 mg/kg plus 12 mg/kg of benserazide intraperitoneally once a day for 7 weeks	Sham-operated animals with intact nucleus accumbens core plasticity reliably developed food-addiction–like behavior when exposed to intermittent access to a highly palatable food 6-OHDA–lesioned animals displayed no increasing interest about the chocolate, but the unresponsiveness was rescued by L-dopa Food-addiction–like behavior relies on an intact ventral striatum core

#### Human case–control studies

One study [48] applied the PET technique among ten patients with obesity and BED and eight controls with obesity but not BED. PET scanning with [^11^C]raclopride was conducted to measure extracellular dopamine release in response to food or neutral stimulation, after placebo or after oral methylphenidate (a dopamine transporter inhibitor). Results revealed that under the condition of food stimuli and methylphenidate, binge eaters showed significantly more dopamine release in the caudate compared to non-binge eaters, and the increased dopamine release was significantly correlated with higher binge eating severity.

Davis et al. [58] compared five polymorphisms in the dopamine D2 receptor genes (e.g., Taq1A, C957T, and − 141C ins/del) between 79 adults with obesity and BED and 151 adults with obesity but without BED. The results showed that participants with BED were more likely to carry the A2/A2 allele of Taq1A and T allele of C957T, which suggested that they had greater D2 receptor availability/affinity compared to the controls without BED.

#### Human cross-sectional studies

A research group from Spain analyzed the associations of three polymorphisms in the dopamine genes (D2 receptor: Taq1A; D3 receptor: Ser9Gly; and dopamine transporter: DAT1 variable number of tandem repeats) with binge eating symptoms in patients with BED (n = 34) or BN (n = 80) [60]. Results revealed that BED patients with the Ser9Gly variant (increases the D3 receptor binding affinity for dopamine, 47% of the group) showed more eating disorder-related psychopathology than BED patients with the Ser9Ser variant. Neither of the polymorphisms in the D2 receptor gene (Taq1A) or dopamine transporter gene (DAT1) was associated with symptom severity in the BED group.

In a study with 74 BN patients [62], the authors examined the dopamine D4 receptor polymorphism, a 48-base pair region with a variable number of tandem repeats (alleles are denoted 7R and non-7R, the 7R allele decreases D4 receptor availability and binding affinity). They found that 77% of BN patients carried non-7R/non-7R alleles. In contrast, only 4.1% of BN patients carried the 7R/7R allele. Furthermore, carriers of a combination of the non-7R/non-7R with other D4 receptor alleles that confer personality traits that are risk factors for binge eating (e.g., attention deficit, borderline personality), experienced more severe general psychopathology compared to non-carriers.

In a study of 1003 community-based adults [65], the authors examined the associations between COMT gene polymorphisms and the likelihood of binge eating symptoms. They found that individuals carrying the Met allele of the COMT gene (lower activity allele) were at a higher risk for binge symptoms compared with Val/Val allele carriers.

Davis et al. [59] examined whether a multilocus genetic profile score based on six dopamine-related polymorphisms (e.g., D2 receptor: Taq1A; dopamine transporter: DAT1 variable number of tandem repeats; COMT: Val158Met) was associated with binge eating symptoms in 120 adults. Results showed that a higher multilocus genetic profile score, which reflected higher striatal dopamine signaling, was linked to more binge eating behaviors.

A small-scale study [78] of eight adults with obesity (but without a diagnosed eating disorder) used electroretinography to estimate brain dopamine activity after oral food stimuli. The authors found that the cone electroretinography response significantly increased to food stimuli, and the increased response was positively associated with binge eating symptoms.

#### Animal studies

An animal study [75] used male rats that were either dopamine-depleted (n = 30) or dopamine-intact (n = 15), and the dopamine-depleted rats were further randomized into the saline group (n = 15) and L-DOPA group (n = 15). With the intermittent access paradigm, the rats in the dopamine-intact and dopamine-depleted with L-DOPA groups developed binge eating behaviors; however, the rats in the dopamine-depleted with saline were non-responsive to the procedure. This result led to the conclusion that intact dopamine is necessary for driving binge eating behaviors.

Another animal study [83] used female rats to evaluate whether inhibiting D2 receptors by injecting the antagonist, raclopride, in the nucleus accumbens reduced binge eating behaviors in binge-eating rats. With the intermittent access paradigm, the rats who developed binge eating (n = 16) were given an injection of raclopride (n = 8) or vehicle (n = 8). Results showed that rats with intra-nucleus accumbens raclopride injection demonstrated reduced meal frequency, meal duration, and sucrose solution intake compared to those with vehicle injection. Thus, the availability of dopamine D2 receptors in the ventral striatum is necessary to maintain binge eating behaviors in female rats.

### Studies that reported hypodopaminergic state in binge eating

The hypodopaminergic state (n = 17) is characterized by 1) six case–control studies that reported decreased dopamine levels, reduced dopamine activities, and lower dopamine receptor availability/affinity in patients with binge eating compared to those without binge eating; 2) two cross-sectional studies that showed negative associations between dopamine levels and binge eating symptoms in patients with BN or BED; 3) six RCTs and one randomized crossover study that reported using dopamine synthesis inhibitor triggered binge eating, or using dopamine transported inhibitor reduced binge eating; and 4) two animal studies that demonstrated lower dopamine receptor levels/affinity in binge rats (Table 2).

**Table 2:** Studies that reported hypodopaminergic state in binge eating

Study characteristics	Study purpose	Participant characteristics	Measurement	Results
Frank et al. 52 Case–control	To test the associations between dopamine and learning in BN patients	20 purging type BN female (age: 25.2 ± 5.3; BMI: 22.6 ± 5.7) 23 healthy control, matched for age and level of education (age: 27.2 ± 6.4; BMI: 21.5 ± 1.2) In BN patients, weekly binge episodes 23.5; illness duration: 74.2 months Among the BN patients, 12 have one or more of the following: major depressive disorder, social phobia, anxiety disorder, PTSD	The temporal difference model: individuals learned to associate different taste stimulus (sucrose solution, no solution, and artificial saliva) with a paired conditioned visual stimulus Event-related fMRI: after breakfast (8-9am) BOLD signal	BN individuals had reduced brain response in the ventral putamen, amygdala, insula and orbitofrontal cortex compared to controls for both taste conditions Binge/purge frequency significantly predicted reduced dopamine response in the BN for the left insula, substantia nigra, left amygdala, right amygdala, right insula, left ventral putamen, and right ventral putamen These results strongly suggest reduced dopamine reactivity in BN is related to illness severity
Vaz-Leal et al. 86 Case–control	To compare 24-h urinary excretion of dopamine between BN patients and healthy controls	75 female with purging type BN (age: 22.9 ± 2.7; BMI: 22.5 ± 1.6) 30 healthy female Caucasian controls (age: 23.6 ± 3.3; BMI: 22.0 ± 1.6) All BN patients were severe: the mean of binging at assessment was 1 per day Exclusion: substance abuse	Urinary 24-h excretion of dopamine was quantified using column chromatographic methods	BN patients had significantly lower 24-h urinary excretion of dopamine
Broft et al. 47 Case–control	To assess striatal D2 receptor density and striatal dopamine release in patients with BN	16 BN female: (age: 24.4 ± 5.1; BMI: 21.7 ± 1.4) 17 healthy control female: (age: 24.9 ± 4.2; BMI: 21.4 ± 2.0) BN patients: duration of illness 7.8 years Exclusion: current or past Axis I disorders, diagnosis of current ADHD or past history of anorexia nervosa, alcohol or substance abuse, active suicidal ideation, use of fluoxetine and other psychoactive medications, ongoing medical or neurological illness, pregnancy	PET scanning after a standardized meal Two scans with [11C]raclopride: a baseline scan and a second scan which began 60 min following administration of methylphenidate Eating disorder examination (EDE-12)	The difference in D2 receptor binding potential between the patient and control groups was not statistically significant Low striatal dopamine response to methylphenidate are present at significant levels only in the putamen, but not in the ventral striatum A statistically significant association between striatal dopamine release and frequency of objective binge episodes: the lower the striatal dopamine response to methylphenidate, the greater the frequency of binge eating in the previous 28 days
Majuri et al. 49 Case–control	Compare dopamine function between BED and healthy controls	7 BED female (age: 49.4 ± 5.1; BMI: 30.9 ± 6.6) 17 healthy controls (age: 43.3 ± 11.1; BMI: 24.8 ± 2.1) BN patients: duration of illness 18.1 years None of the subjects were using medications known to have effects on the dopamine system	PET with [18F]fluorodopa [18F]fluorodopa scan at 1430–1530 h after a standardized lunch	A lower dopamine synthesis capacity in BED compared with controls in the nucleus accumbens with the cluster extending to the caudate and putamen BED is characterized by a widespread reduction in striatal dopamine synthesis capacity
Frieling et al. 57 Case–control	To examine the peripheral expression of dopaminergic genes in patients suffering from eating disorders	24 female with BN (age: 25.8 ± 7.7; BMI: 22.6 ± 2.6) Duration of illness: 9.0 ± 5.8 years 30 age-matched healthy women (age: 22.0 ± 4.5; BMI: 21.7 ± 3.7)	Total RNA was extracted from whole frozen EDTA-blood using a standard phenol–chloroform-extraction in Qiazol (Qiagen), followed by column-purification with Rneasy Mini Kit (Qiagen)	BN showed an elevated peripheral expression of dopamine transporter mRNA and a downregulation of the DRD2 expression when compared with the controls
Thaler et al. 61 Cross-sectional	Examine dopamine polymorphisms acting upon postsynaptic receptors in women with bulimia-spectrum eating disorders DRD2 TaqA1 rs1800497, DRD4 7R, COMT rs4680, DAT1	269 bulimic women with full-blown and sub-threshold bulimia (age: 25.9 ± 6.7; BMI: 22.7 ± 3.8) 65.1% BN-purging subtype, 5.2% BN-nonpurging subtype, and 29.7% Eating Disorder Not Otherwise Specified Number of binge episodes per month was 25.82 (SD = 35.71) 52.7% of the sample were using a psychoactive medication	Genomic DNA was extracted from blood leukocytes using the FelxiGene DNA Kit (Qiagen) Eating disorders examination	Participants with the Val/Val genotype had higher levels of binge eating than those with a Met allele Participants with the DAT 10/10 genotype reported higher levels of binge eating than did those with any 9-repeat allele
Corwin et al. 56 Animal model	Test whether dysregulation of dopamine system using dopamine D1 and D2 agonist and antagonist influence binge eating	Male Sprague–Dawley rats A rat model of binge-type eating in which non-food-deprived rats with brief intermittent (3 days/week) access to an optional source of dietary fat binge on the fat relative to rats with brief daily access to the same fat	D1 agonist (SKF 81,297) targeting PFC—M2 region D1 antagonist (SCH 23,390) targeting PFC—M2 region D2 agonist (quinpirole) targeting PFC—M2 region D2 antagonist (eticlopride) targeting the Cg1/M2 Micropunched tissue from the VTA, NA core and shell, central nucleus of the amygdala, and PFC were collected and analyzed for relative mRNA expression using the comparative threshold cycle method	VTA Before binge, gene expression for tyrosine hydroxylase, the dopamine transporter, and the D2-like receptor was higher in the binge rats than the control rats Within intermittent access, tyrosine hydroxylase was significantly higher before binge but returned to control level after binge Gene expression for the D1-like receptor was significantly lower in the intermittent access rats relative to D No difference in amygdala or nucleus accumbens PFC Neither the D1 agonist nor the D1 antagonist infused into the M2 region of the PFC affected shortening intake When the D2 agonist was infused into the M2 region, shortening intake was significantly reduced after the highest dose in both intermittent access and binge eating rats D2 blockade with eticlopride stimulated intake in the intermittent access rats, but was without effect in the control rats Conclusion The initially-elevated VTA dopaminergic gene expression may contribute to binge initiation The PFC, and D2 receptors in particular, functions as a behavioral brake to limit bingeing
Amorim-Barbosa et al. 84 Case–control	Evaluate COMT activity in patients with BN and BED	10 BN 10 BED (BMI: 39.3 ± 1.4) 10 control subjects matched for age and gender (BMI: 22.5 ± 0.9) Individuals treated with antidepressant were analyzed in separate groups Exclusion: history of tabagism and drug consumption	EDE-Q COMT activity was determined by the ability of enzyme preparations to methylate adrenaline to metanephrine in crude homogenates and soluble-COMT	Patients with BN or BED showed increased soluble-COMT activity when compared with controls
Chawla et al. 55 Animal model	Examine the gene expression of dopamine receptors (Drd1, Drd2, Drd4) in binge eating rats	44 male, young adult Sprague–Dawley rats Intermittent access binge group (binge, n = 28), daily access group (DAILY, n = 8) and chow controls (CON, n = 8) Rats in the binge group were further divided into binge eating prone (BEP) and binge eating resistant (BER) categories	Total RNA was extracted from tissue punches using the RNeasy Lipid Tissue Mini kit (QIAGEN)	PFC (OFC, mPFC, FrC) OFC: significantly lower expression of Drd1 in BEP as compared to CON group OFC: significantly lower expression of Drd4 in BEP as compared to CON group mPFC: significantly greater expression of Drd1 in BEP as compared to CON group mPFC: significantly lower expression of Drd4 in BEP as compared to DAILY rats Nucleus accumbens Lower Drd2 expression in BEP rats as compared to CON rats A negative correlation between the average shortening consumed in last week of binge paradigm and the nucleus accumbens Drd2 expression
Heal et al. (2017) Animal model	To investigate the dopaminergic systems (dopamine receptors, dopamine transporter, dopamine concentration) in the brains of binge-eating and control rats	Lean, female Wistar rats BED was induced by intermittent access (Unpredictable intermittent two-hour access to chocolate over a period of 28 days) 20 BED group and 20 non-binge eating control group	Whole brains were removed and striata were dissected before being frozen on dry ice Eight- or 10-concentration radioligand saturation binding analysis was used to measure tissue receptor density and affinity	Striatal D1 receptors was significantly reduced by 38.7% in the binge-eating group (in the caudate putamen, but not nucleus accumbens) Binge-eating did not alter the density or affinity of D2 receptors in the striatum compared with controls Binge-eating did not alter either the density of striatal DAT sites or the affinity of DAT sites in the brains of the rats No significant differences between the concentrations of dopamine in the striatum, frontal cortex or hypothalamus the rate of dopamine turnover were not significantly altered in either the striatum or prefrontal cortex
Gervasini et al. 62 Cross-sectional	Evaluate associations between the Val158Met polymorphism in the COMT gene and general psychopathological symptoms	74 BN female (BMI: 24.6 ± 6.9) 30 BED female (BMI: 34.3 ± 10.2) 51.8% were being treated with antidepressant Exclusion: dementia, mental retardation, schizophrenia, Turner’s syndrome, other neurological disorders and underlying endocrine pathologies	Symptom Checklist 90 Revised Genomic DNA was isolated from peripheral blood leukocytes	BN patients who carried the Val-allele scored higher in all nine scales and three global indices of the Symptom Checklist 90 Revised questionnaire
Grob et al. 76 Randomized, double-blind, placebo-controlled, crossover design	To test dopamine function in remitted subjects with BN who performed a reinforcement-learning task after catecholamine depletion	19 women who had BN remission (age: 25.2 ± 3.5; BMI: 21.7 ± 2.9) 28 healthy control women (age: 25.8 ± 3.6; BMI: 22.1 ± 2.1) Exclusion: lifetime diagnosis of psychosis, major medical or neurological illness, psychoactive medication exposure in the past 6 months, lifetime history of substance dependence, pregnancy, suicidal ideation	Catecholamine depletion achieved by oral administration of alpha-methyl-paratyrosine (AMPT) Thirty hours after the first AMPT/placebo administration, participants completed a 25-min probabilistic reward task Examination-Questionnaire (EDE-Q) was assessed at (0, 26, and 30 h after the first AMPT/placebo administration) and on the 3 subsequent days (54, 78, and 102 h after the first AMPT/placebo administration)	Following catecholamine depletion, rBN subjects (but not controls) showed reduced responsiveness to rewards leading to an inability to modulate behavior as a function of reinforcement history This DA-mediated deficit was not associated with time in remission from BN, suggesting that reduced reinforcement learning might represent a stable, trait-like feature of BN
Grob et al. 77 Randomized, double-blind, placebo-controlled, crossover design	To examine the effect of catecholamine depletion on bulimic symptoms in remitted BN and controls	18 female who had been in remission from BN for at least 6 months (age: 25.6 + 4.7; BMI: 21.2 + 1.7) 31 controls who had no history of any psychiatric disorder (age: 25.8 + 3.8; BMI: 22.4 + 2.2) Exclusion: current Axis I psychiatric disorders, a lifetime diagnosis of psychosis, major medical or neurological illness, psychoactive medication exposure within the previous 6 months, lifetime history of substance dependence, pregnancy, suicidal ideation	German Version of the Eating Disorder Examination—Questionnaire Measurements were conducted immediately before the first AMPT or placebo intake (prechallenge) and 26, 30, 54, 78, 102 h after the first AMPT or placebo administration The time frame was divided into the controlled environment (time points 26 and 30 h) and the uncontrolled environment (time points 54, 78, and 102 h)	Under controlled environment, rBN subjects reported more bulimic symptoms in the conditions in which they received AMPT compared with the placebo condition The results indicate that catecholamine depletion induced a transient reappearance of mild eating disorder symptoms in remitted subjects with a history of BN, which is in line with the desensitized dopaminergic theory
Guerdjikova et al. 79 12-week randomized, double blind, parallel-group, and flexible-dose study	To evaluate the effect of Lisdexamfetamine dimesylate (LDX) on binge eating	50 adults (92% women; age: 37.7 ± 8.9; BMI: 39.8 ± 9.3) who display ≥ 3 binge eating days/week were randomized to LDX (n = 25) or placebo (n = 25) Mean baseline weekly binge eating days/week was 4.2 (1.2); binge eating episodes/week was 5.1 (3.1) Exclusion: current anorexia nervosa or bulimia nervosa, current suicidal ideation, receipt of a psychological or weight loss intervention for BED, substance use disorder, a lifetime history of psychosis, mania, or hypomania; a clinically unstable medical illness; receipt of psychotropic medication	Participants were randomized to receive LDX or placebo in a 1:1 ratio Participants were evaluated at least twice during the screening period; after 1, 2, 3, 4, 6, 8, 10, and 12 weeks during the treatment period; and 1 week after study medication discontinuation	Comparing the baseline-to-endpoint change score differences, LDX was associated with statistically significant decreases in binge eating days/week, binge eating episodes/week
McElroy et al. 80 Randomized, double-blind, parallel-group, placebo-controlled clinical trial	To examine the efficacy and safety of lisdexamfetamine dimesylate to treat moderate to severe BED	259 patients with moderate-to-severe BED (81.5% female; age: 38.7 ± 10.2; BMI: 34.9 ± 5.3) Exclusion: current bulimia nervosa, anorexia nervosa, ADHD, or another psychiatric disorder; a lifetime history of bipolar disorder or psychosis; psychological or weight-loss interventions; use of a psychostimulant; cardiovascular disease; substance abuse; antipsychotics, antidepressants	Participants were randomized (1:1:1:1) to receive placebo or 30, 50, or 70 mg/d of lisdexamfetamine dimesylate Changes in binge eating behaviors were measured at week 11	At week 11, lisdexamfetamine dimesylate treatment with 50 and 70 mg/d, but not 30 mg/d, demonstrated a significant decrease (compared with placebo) in weekly binge eating days per week and binge eating episodes per week
Mueller et al. 53 Double-blind, crossover design	To identify the role of dopamine dysfunction and its relation to behavioral and neural reward-effort integration in bulimia nervosa	17 female participants in remission from BN (rBN, at least 4 months) (age: 29.6 ± 8.9; BMI: 21.6 ± 2.3) 21 female healthy volunteers (age: 27.3 ± 9.4; BMI: 24.2 ± 3.3) Did not exclude major depression, using psychological medications	Participants received once catecholamine depletion induced by alpha-methyl-paratyrosine (AMPT) and once sham depletion in a randomized order During fMRI, participants performed monetary incentive delay (MID) task The monetary earning in this task indicates the effectiveness of integrating reward magnitudes and effort costs to guide behavioral performance	Healthy controls earned less money (reduced the ability to integrate effectively reward magnitudes and effort costs to guide behavioral responses) following AMPT relative to the sham condition, whereas the monetary earning of rBN participants was not influenced by AMPT The reward–effort integration indicated by the monetary earning was found to be already reduced in rBN participants in the sham condition, the longer the duration of active illness, the less money they earned in the sham condition
Grilo et al. 81 12-week double-blind, parallel-group treatment	Evaluate the efficacy and safety of two fixed doses of dasotraline (4 and 6 mg/d) in adults with BED	485 moderate-to-severe BED patients (female: 83.9%; age: 37.6; BMI: 34.5) Moderate-to-severe BED: based on a history of ≥ 2 binge eating days per week for ≥ 6 months prior to screening; and patient diary-confirmed criteria of ≥ 3 binge eating days per week for each of the 2 weeks prior to study baseline Exclusion: lifetime history of bulimia nervosa or anorexia nervosa; initiation of a formal weight loss program; a lifetime history of psychotic disorder, bipolar disorder, hypomania, or ADHD; history of moderate-to-severe depression; use of antidepressants, psychostimulants, or mood stabilizers; a history of substance abuse; diabetes, hypertension or cardiovascular disease	Participants were randomized to receive 4 mg/d dasotraline, 6 mg/d dasotraline, or placebo Changes of binge eating were measured at week 12	At week 12, treatment with dasotraline was associated with significant improvement in number of binge eating days per week on the dose of 6 mg/d Dasotraline treatment improved obsessional thoughts related to binge eating and ruminative preoccupations that interfered with daily functioning, and reduced the compulsion to binge eat, increasing patient control and ability to resist the binging urges

#### Human case–control studies

Frank et al. [52] used fMRI to examine the brain activity in 20 females with BN and 23 healthy controls in a temporal difference model during which participants learned to associate three unconditioned taste stimuli to a paired conditioned visual stimulus. In healthy subjects, it is expected that dopamine levels will increase in response to unexpected unconditioned stimuli, and dopamine levels will decrease if the conditioned stimuli are followed by an omission of the unconditioned stimuli. Results showed that BN individuals had a blunted BOLD response (a proxy of dopamine function) to both unexpected unconditioned stimuli and omission of unconditioned stimuli in several brain areas (e.g., ventral putamen, orbitofrontal cortex) compared to controls. Furthermore, the reduced response was significantly correlated to binge/purge frequency.

A small-scale study [49] used PET scanning with [^18^F]fluorodopa to compare striatal dopamine synthesis in seven adults with binge eating and obesity and 17 healthy-weight controls. Results revealed a 20% lower dopamine synthesis capacity in the nucleus accumbens in the BED group compared to the control group.

Another study [47] conducted PET scanning with [^11^C]raclopride among 16 BN patients and 17 healthy controls. The authors found that the BN group had a blunted dopamine release to methylphenidate in the putamen, and the blunted release was correlated to a greater frequency of binge eating in the previous 28 days.

Frieling et al. [57] compared the peripheral expression, which is believed to be somewhat reflective of brain status, of dopamine D2 receptor, dopamine D4 receptor, and dopamine transporter genes in 24 patients with BN and 30 healthy controls. This study reported a downregulated mRNA expression of the D2 receptor gene and an elevated mRNA expression of dopamine transporter (which would result in less dopamine being available in synapses) in the BN group compared to controls.

One study analyzed the soluble COMT in erythrocytes with ten BN, ten BED, and ten controls [84]. Soluble COMT is an isoform of COMT that is highly expressed in peripheral tissues, and its activity is encoded for the COMT gene. This study found that participants with BED or BN had significantly higher soluble COMT activity compared to controls, which collaborate other studies that reported high-activity allele of COMT (Val-allele) in binge eating.

Another study also used peripheral levels of dopamine to infer its central activities. This study compared the urinary levels of dopamine between 75 female patients with purging BN and 30 healthy controls [86], and found lower 24-h excretion of dopamine in patients with BN.

#### Human cross-sectional studies

Thaler et al. [61] examined the relationship between the polymorphisms of dopamine-regulating genes (dopamine transporter, COMT) and binge eating among 269 women with spectrum-bulimia disorder. The authors found a positive relationship between dopamine transporter 10-repeat allele (associated with higher dopamine transporter expression) and higher levels of binge eating, and that women with a COMT Val/Val genotype (degrades dopamine at a faster rate, resulting in lower dopamine levels) had higher levels of binge eating than did those with a Met/Met genotype.

Likewise, a cross-sectional study [64] of 303 patients with eating disorders (199 with AN, 74 with BN, and 30 with BED) found that BN patients who carried the Val-allele of COMT gene had more severe psychopathology compared to BN patients who carried the Met allele, although no such association was found for the BED patients.

#### Human RCTs and randomized crossover study

A clinical trial [77] randomized 18 women with remitted BN and 31 control women to receive alpha-methyl-para-tyrosine (a dopamine synthesis inhibitor) or placebo for at least seven days. Results showed that subjects with remitted BN reported more bulimic symptoms in the alpha-methyl-para-tyrosine condition compared with the placebo condition. Two other studies [53, 76] from the same research group further demonstrated that subjects with remitted BN had blunted reward responsiveness not only after [76] but also before administration of alpha-methyl-para-tyrosine [53], suggesting a hypodopaminergic state in remitted BN patients.

McElroy et al. [80] conducted a multi-site study to test the efficacy of lisdexamfetamine dimesylate (a dopamine transporter inhibitor) to treat moderate-to-severe BED. Participants were randomized to receive placebo (n = 63) or 30 mg/d (n = 66), 50 mg/d (n = 65), or 70 mg/d (n = 66) of lisdexamfetamine dimesylate. Results showed that at Week 11, participants treated with 50 and 70 mg/d lisdexamfetamine dimesylate demonstrated a significant decrease in weekly binge eating frequency compared to the control group. The results were replicated in another RCT [79], which showed significant decreases in binge eating frequency among adults with obesity and binge eating who received lisdexamfetamine dimesylate treatment compared to the placebo group at one week after drug termination.

A 12-week RCT [81] evaluated the effect of dasotraline, a less specific serotonin-norepinephrine-dopamine transporter inhibitor, in the treatment of BED. Patients (n = 491) who displayed moderate-to-severe BED were randomized to a 4 mg/d dasotraline group, a 6 mg/d dasotraline group, and a placebo group. Results revealed that treatment with 6 mg/d dasotraline significantly reduced weekly binge eating frequency and improved BED-related symptoms at week 12.

#### Animal studies

One animal study [55] divided 44 male rats into three groups: intermittent access binge group (n = 28), daily access group (n = 8), and chow controls (n = 8). The rats in the binge group were further classified into binge eating prone (n = 8), binge eating neutral (n = 12), and binge eating resistant (n = 8) groups. Authors found lower D1, D2, and D4 receptor mRNA expression in several brain areas (e.g., nucleus accumbens, orbitofrontal cortex) in binge eating prone rats compared to control rats. Additionally, there was a negative correlation between the D2 mRNA expression in nucleus accumbens and food consumed in the binge rats.

Corwin et al. [56] tested whether activating or inhibiting D1 and D2 receptors in the prefrontal cortex altered the binge eating behaviors of rats. Binge eating was achieved by intermittent access to high-fat shortening, and the authors found that although neither the injection of D1 agonist nor the D1 antagonist into the prefrontal cortex affected shortening intake, the D2 agonist and D2 antagonist significantly reduced and stimulated intake in binge-eating rats, respectively.

### Studies that reported unchanged dopaminergic state in binge eating

The unchanged dopaminergic state (n = 5) is characterized by 1) three case–control studies that reported no difference of dopamine receptor availability/affinity in patients with binge eating compared to those without binge eating; 2) an RCT that showed no effect of dopamine receptor antagonist on binge behaviors; and 3) an animal study that demonstrated no effect of dopamine receptor agonist or antagonist on binge behaviors (Table 3).

**Table 3:** Studies that reported unchanged dopaminergic state in binge eating

Study characteristics	Study purpose	Participant characteristics	Measurement	Results
Yilmaz et al. 66 Case–control	Examine if certain variants of the COMT genetic markers (rs6269, rs4633, rs4818 and rs4680) are more common in BN versus controls	240 women with purging subtype BN (age: 26.0 ± 7.0; BMI: 22.2 ± 3.4) Ethnicity-matched female controls Among BN patients, 20 had ADHD Exclusion: a maximum lifetime BMI ≥ 35 kg/m2, history of a psychotic episode, history of bipolar disorder, diabetes, thyroid or endocrine disorder	Eating disorder examination Blood lymphocyte DNA was extracted using the high-salt method	There were no differences between bulimic women and nonpsychiatric controls in terms of genotype, allele, and haplotype frequencies for any of the four COMT markers COMT Val158 allele was overrepresented and the medium-activity haplotype was underrepresented in BN with childhood ADHD history
Yilmaz et al. 63 Case–control	To compare DRD4 hypofunctional allele frequencies in BN when compared with controls	157 female with purging type BN (age: 26.0 ± 7.1; BMI: 22.1 ± 3.2) 157 ethnicity-matched female controls Among BN patients, 19 had ADHD	Eating disorder examination Blood lymphocyte DNA was extracted using the high-salt method	There were no differences between BN probands and controls in terms of DRD4 allele frequencies 34.2% of BN probands with childhood ADHD carried at least one copy of 2R or 7R allele. In contrast, only 14% of BN probands who did not have childhood ADHD carried one or both alleles
Groleau et al. 85 Case–control	Examine the associations between DRD2 methylation and bulimic eating disorder	Of the 52 women with a bulimia spectrum disorder (age: 24.7 ± 5.7; BMI: 22.8 ± 4.4), 63.5% BN-Purging subtype, 3.8% for BN-Non Purging subtype, and 32.7% an Eating Disorder Not Otherwise Specified 67.3% were using a psychoactive medication, 8 had bipolar disorder, 14 had childhood sexual abuse, 23 had childhood physical abuse 19 female controls without childhood maltreatment (age: 23.7 ± 4.6; BMI: 22.4 ± 2.8)	Eating disorder examination The sequence of DRD2 was identified using UCSC Genome Browser Assembly February 2009	No overall difference as to DRD2 methylation between non-eating disorder and bulimia spectrum disorder groups Bulimia/Borderline Personality Disorder group had a significantly higher mean methylation than did either Bulimia/no-Borderline Personality Disorder or no eating disorder groups Bulimia/Childhood Sexual Abuse women have a significantly higher mean methylation than did No Eating Disorder women
Dodds et al. 54 Randomized, double-blind, placebo-controlled, two-way cross over design	Investigate the effects of the selective dopamine D3 receptor antagonist GSK598809 on brain activation to food images in a sample of binge and emotional eating obese and overweight subjects	26 obese participants who reported binge eating behaviors and emotional eating (15 male, 11 female; age: 35.1 ± 7.1; BMI: 32.7 ± 3.7) Minimum 1 episode/week binge eating behavior Had no personal or family history of psychiatric disorders, had no history of substance abuse, had no history of eating disorders, had reported no significant weight loss (or gain)	Participants received either GSK598809 (175 mg capsule) or placebo Brain activities to high-fat or general food images were measured by fMRI, which was performed approximately 3 h post dose	No significant effect of GSK598809 on activation to food images or to high calorie food images in any of the brain regions: amygdala, insula, ventral striatum, caudate, putamen, midbrain and hypothalamus The effect of GSK598809 on brain activation to food images, or more specifically to high calorie food images, did not correlate significantly with scores on any of the personality/eating behavior questionnaires There was no effect of GSK598809 on subjective feelings of hunger and craving
Lardeux et al. 82 Animal study	Test whether injection of dopamine receptor antagonists into the accumbens reduced consumption of a sweet high-fat liquid in rats with and without a history of intermittent binge access to the liquid	Male Long–Evans rats Rats were divided in three group, the intermittent access (binge) group (n = 93) and two control groups: the water access group (n = 83) and the continuous access group (n = 38)	Rats received injection of vehicle and dopamine D1 or D2 receptor antagonist	The injection of dopamine D1 and D2 receptor antagonist in the nucleus accumbens core or shell did not impact the consumption of food in any groups

#### Human case–control studies

In one study that included 206 women with a full threshold or subthreshold BN and 102 healthy controls [85], the authors analyzed the methylation (an epigenetic modification that can impact gene transcription and expression) of the dopamine D2 receptor gene, but found no difference between the two groups. However, it is worth mentioning that 45 of the participants in this study had bipolar disorder or childhood abuse, and the D2 receptor DNA methylation was significantly higher in those participants compared to those without bipolar disorder or childhood abuse.

In another case–control study of 240 females with BN and 240 controls, the authors did not find any difference between BN patients and controls in terms of D4 receptor gene polymorphisms [63] or COMT allele frequencies [66]. However, a subgroup of BN patients had a history of attention deficit hyperactivity disorder, which could bias the results.

#### Human RCTs

In an RCT [54] with 26 overweight and obese participants who reported binge eating behaviors, the participants were given either a dopamine D3 receptor antagonist or placebo and were exposed to high-fat and general food images. However, fMRI results showed that brain activation (e.g., ventral striatum, caudate, putamen) to food images was not modulated by the D3 receptor antagonist. Additionally, the D3 receptor antagonist had no effect on self-reported eating behaviors.

#### Animal studies

An animal study [82] did not find any effect of injecting dopamine D1 (SCH23390) or D2 receptor antagonists (raclopride) into the accumbens on food consumption in male rats with or without a history of intermittent binge access to palatable foods.

## Discussion

This study reviewed the role of dopamine, including dopamine levels, dopamine activities, dopamine regulator levels/activities, and dopamine receptor availability/affinity, in relation to binge eating among both humans and animals. The primary finding is that the majority of studies (26/31, 83.9%) documented an altered dopaminergic state related to binge eating. However, the literature is inconsistent concerning the direction of the alteration, supporting either a hyperdopaminergic (9/26, 34.6%) or a hypodopaminergic (17/26, 65.4%) state in binge eating.

The dissonance may be partially due to the complexity of the dopamine system (e.g., bursting vs. phasic release) [86] and the variability in sample characteristics (e.g., weight status, age, race), study design, diagnosis criteria, and neuroimaging/genetic/pharmacological techniques, making the interpretations of study findings less straightforward. Additionally, the potential confounders that may influence the reward system and dopamine function (e.g., psychiatric diseases, medications, history of dietary restraint, hunger/satiety) may also contribute to the heterogeneity. In this review, although most studies excluded individuals with psychiatric diseases (e.g., major depression symptoms, post-traumatic stress disorder) and people who had a history of childhood abuse, other studies included these individuals [52, 61, 63, 66, 85]. Because individuals with depression and post-traumatic stress disorder usually have diminished dopamine function [87, 88], and those with childhood abuse usually have elevated dopamine function [89], the inclusion of people with these comorbidities may bias the results.

Another reason that can possibly explain the inconsistency is that this review included two distinct types of eating disorders: BED and BN. Although both BED and BN are marked by binge eating symptoms, BN additionally requires using compensatory behaviors after binge episodes (e.g., vomiting, using laxatives). Notably, the few studies that have compared the etiology and neural underpinnings of BED and BN revealed that despite a large magnitude of overlap [90], differences exist in the severity of dopamine-related risk factors. For example, patients with BED have been shown to have higher reward sensitivity and less intense top-down control to inhibit the increased food craving than those with BN [91, 92], suggesting the possibility that dopamine may function differently in BED and BN. In this review, among the eight studies that reported an altered dopaminergic state in the BED population, one half of the studies supported the hyperdopaminergic state and the other half supported a hypodopaminergic state but with a stronger level of evidence from RCTs. A more consistent trend was observed in the BN population with nine out of ten studies supporting a hypodopaminergic state, suggesting that dopamine may be downregulated in this specific eating disorder. Future studies may benefit from directly comparing the dopamine function among BED, BN, and preferably purging-only groups to better understand how dopamine contributes to binge-related eating disorders.

Although there are possible explanations for the inconsistent findings, two hypotheses that could potentially reconcile the inconsistency should be considered in future studies. The first hypothesis is that instead of being mutually exclusive, the hyperdopaminergic and hypodopaminergic states may co-exist, but in different stages of binge eating. This hypothesis is supported by the observations in this review as well as similar conjectures proposed in other disease conditions that are highly correlated with binge eating, such as obesity and substance use disorder.

Among studies included in this review, although not all of them reported the stage or severity of binge eating, the two studies [59, 78] conducted among community-based adults who were completely or partially free of diagnosed binge eating supported a positive association between dopamine activity and binge eating symptoms. In contrast, eight studies [47, 49, 52, 57, 61, 79–81, 93] that included participants with moderate-to-severe binge eating or participants with a long illness duration (three being RCTs) supported a hypodopaminergic state in binge eaters. Plus, the three studies that documented a desensitized dopamine system in remitted BN patients [53, 76, 77] provided further evidence of a hypodopaminergic state in the late stages of binge eating. These results suggest that dopamine elevations may contribute to the initiation of binge eating, but a downregulation may occur after repeated bingeing, which perpetuates the behavior. Notably, while no human study has longitudinally examined dopamine function over the course of binge eating, one animal study has investigated the overall neural activation in the nucleus accumbens among female rats in early- and chronic- stages of binge eating [94]. The study results suggested a hyper-neural activation to reward in the early stages of binge eating and a decreased activation in the later stages of binge eating [94].

The hypothesis of hyper- then hypodopaminergic state in binge eating is also consistent with the reward-related models or theories in obesity (dynamic vulnerability model), drug addiction (dopamine desensitization theory), and alcohol use disorder (three-stage model). In obesity literature, two opposing dopamine-related theories are debated—the reward surfeit and reward deficit theories [95]. The former posits that greater reward responsivity (greater dopamine signaling) to high-energy foods increases the risk for obesity, while the latter proposes the opposite [96, 97]. Stice et al. [96] reviewed prospective studies that examined predictors of weight gain and found convergent evidence supporting an association between greater reward responsivity to high-calorie foods and increased risk for future weight gain. On the contrary, there was little evidence supporting such association for decreased reward responsivity. Therefore, hypo-responsivity of reward is likely to represent the consequence rather than a precursor of weight gain [96].

Binge eating is also highly correlated with substance use disorder as the two disease conditions have a high comorbidity (e.g., binge eaters are more likely to use alcohol and illicit drug compared to controls) [98, 99] and share common symptomatology (e.g., an overwhelming desire for food/substances, a feeling of “loss of control” even in the face of adverse consequences, and preoccupation with thoughts of food/substances) [100], risk factors (e.g., increased reward sensitivity, impulsivity, and diminished self-control), and neurobiological underpinnings (e.g., interruptions in the dopaminergic pathways) [99, 100]. Therefore, the dopamine-related theories of substance use disorder may also apply to binge eating.

The dopamine desensitization theory of drug addiction proposes that dopamine elevations occur in the initial stages of addiction but not after repeated excessive intake of drugs. Instead, the later stage of drug use is possibly associated with a decreased dopamine release, reduced dopamine D2 receptor availability, and downregulated dopaminergic responses to drug cues [101–103]. Likewise, the three-stage model (binge/intoxication, withdrawal/negative affect, and preoccupation/anticipation) of alcohol dependence proposes that the binge/intoxication stage, which is characterized by positive reinforcement processes and is dependent on dopamine release in the right ventral striatum, lays the groundwork for initial transition to addiction. Following chronic alcohol exposure, the subsequent withdrawal/negative affect stage is associated with compromised dopamine functions that contribute to the decreased sensitivity to rewards and alcohol tolerance [104, 105].

An alternate hypothesis to reconcile the inconsistent findings is that the hyper- and hypodopaminergic states represent two distinct pathways to binge eating, and individual genotypes determine whether it is the hyper- or hypodopaminergic state that confers a risk of binge eating [106]. This hypothesis was initially proposed in the obesity research, and two studies from this review appeared to provide support for distinct pathways.

As previously mentioned, both reward surfeit and reward deficit theories exist to explain the development of obesity. Despite that evidence overwhelmingly supported the reward surfeit theory, Stice et al. [97] tested whether the Taq1A (dopamine D2 receptor gene) polymorphism moderated the relationship between reward responsivity and body fat gain. The authors conducted a food reward fMRI paradigm with milkshake as a stimulus among 153 adolescents, and found that elevated caudate response to milkshake receipt predicted body fat gain for youth with a genetic propensity for the Taq1A A2/A2 allele (higher levels of D2 receptor availability and binding affinity), but lower caudate response predicted body fat gain for youth with a genetic propensity for the Taq1A A1 allele over a 3-year follow-up [97]. This finding suggested that there were distinguishable subtypes of obesity that can be predicted by dopamine genotypes.

In this review, two studies have compared the Taq1A A1 and A2 allele between BED patients and healthy controls, with one study reporting overrepresentation of Taq1A A1 [58] and the other reporting overrepresentation of Taq1A A2 allele [60] in the BED group. This inconsistency could be potentially solved by the possibility that both hyperdopaminergic and hypodopaminergic state predicts binge eating, but in individuals with a genetic predisposition to higher and lower dopamine signaling, respectively. However, this hypothetical interaction between individual genotype and dopamine function should be directly examined in future binge eating studies.

This review has several limitations. First, results were organized based on different dopaminergic states in binge eating behaviors. While this structure makes the opposing viewpoints stand out and emphasizes the possibility of the co-existence of hyper- and hypodopaminergic states in binge eating, it may complicate the direct comparisons between studies that used the same technique (e.g., neuroimaging, genetic) or adopted a similar study design (e.g., case–control, RCT). Additional limitations are mostly limitations that are inherent in the included studies, including small sample size, lack of control for confounding variables in some studies, and the absence of longitudinal studies that cover different stages of binge eating (e.g., onset, maintenance, remission). Moreover, it should be noticed that only a few studies reported the specific brain areas where dopamine alterations occur, and no study functionally correlated the alterations to corresponding risk factors of binge eating. This limitation precludes a clear understanding of what aberrant dopamine functions contribute to the initiation or maintenance of binge eating. Studies from substance use disorder have provided useful clues for the delineation of specific brain areas and dopamine functions in different disease stages. For example, the three-stage model of alcohol dependence elaborates that the three stages—binge/intoxication, withdrawal/negative affect, and preoccupation/anticipation—map onto disturbances in three major neurocircuits (basal ganglia, extended amygdala, and frontal cortex, respectively), which correspond to three functional domains characterized by excessive incentive salience/habit formation, negative emotional states, and dysregulation of executive function [107–109]. Given the correlation between substance use and binge eating, this three-stage model implies the potential to further characterize dopamine function in the process of binge eating, which would ultimately facilitate the prevention and treatment of this problematic eating behavior.

## Conclusion

In conclusion, although most studies have supported altered dopamine levels, dopamine activities, dopamine regulator levels/activities, or dopamine receptor availability/affinity related to binge eating, the direction of the alteration is unclear. Future studies may benefit from a careful control of confounding variables that may influence dopamine functioning (e.g. psychiatric diseases). Furthermore, longitudinal studies are needed to test whether there is a shift from hyperdopaminergic to hypodopaminergic state as binge eating progresses and whether individual genotypes modulate the relationship between dopamine and binge eating, which are two hypotheses that may potentially reconcile the inconsistent findings.

## Data Availability

N/A.
